# Association of Angiotensin-Converting Enzyme Insertion/Deletion Polymorphism with Recurrent Pregnancy Loss: a Meta-Analysis of 26 Case-Control Studies

**DOI:** 10.1055/s-0038-1672137

**Published:** 2018-10

**Authors:** Fereshteh Aslbahar, Hossein Neamatzadeh, Razieh Sadat Tabatabaiee, Mojgan Karimi-Zarchi, Atiyeh Javaheri, Mahta Mazaheri, Elnaz Foroughi, Rezvan Nasiri

**Affiliations:** 1 Department of Obstetrics and Gynecology, Semnan University of Medical Sciences, Semnan, Iran; 2 Mother and Newborn Health Research Center, Shahid Sadoughi University of Medical Sciences, Yazd, Iran; 3 Department of Medical Genetics, Shahid Sadoughi University of Medical Sciences, Yazd, Iran; 4 Department of Obstetrics and Gynecology, Shahid Sadoughi University of Medical Sciences, Yazd, Iran; 5 Department of Pediatric Dentistry, Arak University of Medical Sciences, Arak, Iran

**Keywords:** miscarriage, pregnancy loss, angiotensin-converting enzyme, polymorphism, meta-analysis

## Abstract

**Objective** Previous studies investigating the association between angiotensin-converting enzyme (ACE) gene insertion/deletion (I/D) polymorphism and recurrent pregnancy loss (RPL) risk has provided inconsistent results. The aim of our study was to assess the association between the ACE I/D polymorphism and risk of RPL.

**Methods** All studies published up to January 30, 2018 on the association of ACE I/D polymorphism with RPL were identified by searching the PubMed, Web of Knowledge, and Google scholar databases.

**Results** A total of 26 case-control studies with 3,140 RPL cases and 3,370 controls were included in the meta-analysis. Overall, there was a significant association between ACE I/D polymorphism and RPL risk under the allele model (I versus D: odds ratio [OR] = 0.538, 95% confidence interval [CI] = 0.451–0.643, p ≤ 0.001), the homozygote model (II versus DD: OR = 0.766, 95% CI = 0.598–0.981, *p* = 0.035) and the recessive model (II versus ID + DD: OR = 0.809, 95% CI = 0.658–0.994, *p* = 0.044). Subgroup analysis by ethnicity showed that there was a significant association between ACE I/D polymorphism and increased risk of RPL in Caucasian and West-Asian populations, but not in East-Asians. When stratified by number of recurrent miscarriages (RMs), a significant association between ACE I/D polymorphism and increased risk of RPL was detected in the group of studies with ≥ 2 RMs, but not in studies with ≥ 3 RMs.

**Conclusion** The meta-analysis suggests that ACE I/D polymorphism is associated with increased risk of RPL. The ACE I/D polymorphism may be a risk factor for RPL in Caucasian and West-Asian populations, but not in East-Asians.

## Introduction

Recurrent pregnancy loss (RPL) is a surprisingly common occurrence, which traditionally is defined as 3 or more (≥ 3) consecutive miscarriages before 20 weeks of pregnancy.^1^ ^2^ However, this definition is not used consistently, and pregnancy losses at higher gestational ages are also, in some literatures, classified as miscarriage instead of stillbirth or preterm neonatal death.^3^ It has been estimated that 1 to 3% of couples suffer from recurrent miscarriages.^4^

The etiology of RPL is still unclear and there is a lot of controversy regarding its diagnosis and treatment.^5^ It is well documented that RPL is a multifactorial disorder involving the interaction of genetic, maternal and environmental factors.^6^ ^7^ An increasing number of genetic association studies are performed to determine the genetic background of RPL.^8^ To date, more than 30 putative major genes involved in immunity (PP14, Annexin II, PIBF, and HLA-DRB1) and angiogenesis (TGF-β, VEGF, TIMP-1, MMP-2, MMP-9, ACE) genes for RPL have been identified.^9^ ^10^ ^11^

Angiotensin I-converting enzyme (ACE) is a zinc metallopeptidase that converts angiotensin I to angiotensin II.^12^ The ACE plays an important role in the modulation of vascular homeostasis, inflammation and angiogenesis.^13^ Based on its biological functions, the insertion/deletion (I/D) polymorphism of the ACE gene can be seen as a candidate locus for RPL.^14^ Angiotensin I-converting enzyme is related with plasminogen activator inhibitor-1 (PAI-1) activity, which is a key regulator in embryo implantation.^15^ The human ACE gene is located on chromosome 17q23, and it consists of 26 exons and 25 introns.^16^ ^17^ One of the well-known polymorphisms in the ACE gene is the 287-bp insertion/deletion (or named repetitive element) in intron 16 (ACE I/D).^15^ ^16^ Angiotensin converting enzyme I/D polymorphism (dbSNP rs4646994) is actually not a single nucleotide polymorphism (SNP) at all; instead, it is an I/D of an Alu repeat sequence in an intron of the ACE gene. The ACE I/D polymorphism is associated with ACE activity and levels in plasma and tissues.^17^ ^18^ It has been shown that individuals with the D allele have higher serum ACE activity than those with the I allele. Therefore, ACE enzyme activity is increased in homozygotes for the DD genotype, intermediate in heterozygotes (ID) and decreased in homozygotes for I (II).^19^ Emerging evidence has shown that the ACE D allele leads to increased expression of PAI-1, which can increase the risk of thrombotic events and enhances the production of angiotensin II from angiotensin I.^16^ ^17^

Accumulating studies have assessed the association between this polymorphism and RPL, but the results are unconvincing and unreliable, which may be partly due to the relatively small samples and different ethnic subgroups. The most recent meta-analysis was conducted in 2013, and it aimed at investigating the association of ACE I/D polymorphism with the risk of RPL.^16^ When taking into account the 11 eligible case-control studies, the results indicate that ACE I/D polymorphism is significantly associated with the risk of RPL in the overall population, but not in Caucasian and non-Caucasian populations. In the past 2 years, several other case-control studies conducted to evaluate the effect of ACE polymorphism on RPL provided some new data and diverse conclusions.^15^ ^19^ ^20^ ^21^ ^22^ ^23^ ^24^ Accordingly, we performed the present systematic review and meta-analysis with more eligible studies to investigate the association between ACE I/D polymorphism and the risk of RPL.

## Methods

### Search Strategy

A systematic search of eligible studies on the association of ACE I/D polymorphism with RPL susceptibility was conducted in PubMed, Web of Science, the Chinese Biomedical Literature database (CBM), and the Chinese National Knowledge Infrastructure database (CNKI) up to the January 30, 2018. The following terms were included in the search: (*Recurrent pregnancy loss* OR *recurrent miscarriage* OR *habitual abortion* OR *miscarriage* OR *fetal loss* OR *RPL*) AND (*Angiotensin-converting enzyme* OR *ACE* OR *SERPINE1*) AND (*insertion/deletion polymorphism* OR *I/D polymorphism* OR *rs4646994*) AND (*single nucleotide polymorphisms* OR *SNPs* OR *polymorphism* OR *mutation* OR *variant* OR *genotype*). To minimize potential publication bias, the extracted publications were not limited to English. Additionally, the references list of the retrieved case-control studies, previous meta-analysis, review articles and clinical trials were manually searched for more additional original articles. If there were multiple reports of the same study or overlapping data, only the study with the largest sample sizes or the most recent one was included to the current meta-analysis.

### Inclusion and Exclusion Criteria

Studies were included based on the following criteria: (1) only full-text and published studies; (2) studies with case-control or cohort design; (3) a study evaluated the association of ACE I/D polymorphism with RPL risk; (4) available genotypes frequencies of ACE I/D polymorphism were provided to estimate the odds ratios (ORs) with 95% confidence intervals (CIs). The exclusion criteria were as follows: (1) the study was not conducted on RPL; (2) studies on secondary causes for RPL; (3) family-based linkage studies; (4) abstracts, case reports, and review articles; (5) studies with only RPL cases (not including healthy individuals); (6) studies on the other polymorphisms of the ACE gene; (7) studies without detail genotype frequencies, which were unable to calculate ORs; and (8) duplicate publications of data from the same study.

### Data Extraction

Two authors independently extracted the following data from each eligible study according to the inclusion criteria: first author, year of publication, country of origin, ethnicity, total number of cases and controls, the frequencies of genotypes, minor allele frequencies (MAFs), and Hardy-Weinberg Equilibrium (HWE) test in control subjects. Any discrepancy between these two authors was resolved by reaching a consensus through discussion or the involvement of a third author who made the final decision through discussions.

### Quality Assessment

Two authors performed the quality assessment of the included studies, which was adjusted from the Newcastle-Ottawa scale (NOS) for case-control studies (Table 1), and solved disagreement through discussion. In this scale, six items, including the selection of patients with unexplained recurrent pregnancy loss, source of controls, comparability of cases and controls on the basis of the design or analysis, sample size, quality control of genotyping methods, and HWE, were carefully checked. The quality assessment values ranged from 0 to 10, with higher scores indicating a better quality of the study.

**Table 1:** Scale for methodological quality assessment

Criteria	Score
1. The inclusion and exclusion criteria of patients with unexplained recurrent spontaneous abortion	
Adequate criteria would include experienced clinical and laboratory examinations	2
Inadequate criteria would include the patients' history review as the only evidence	1
Not described	0
2. Source of controls	
Community controls without history of disease	2
Hospital controls	1
Not described	0
3. Comparability of cases and controls on the basis of the design or analysis	
Study controls for selecting the most important factor	2
Patient medical record	1
Not described	0
4. Sample size	
≥ 200	2
100–199	1
< 100	0
5. Quality control of genotyping methods	
Repetition of partial/total tested samples with a different method	1
Repetition of partial/total tested samples with the same method	0.5
Not described	0
6. Hardy-Weinberg equilibrium (HWE)	
Hardy-Weinberg equilibrium in control subjects	1
Hardy-Weinberg disequilibrium in control subjects	0

### Statistical Analysis

The association between ACE I/D polymorphism and RPL was assessed using crude odds ratio (OR) with 95% confidence interval (CI). Five different genetic models were constructed to determine pooled ORs in accordance with the assumed genetic effect of the D allele including: allele model (I versus D), homozygous model (II versus DD), heterozygous model (ID versus DD), dominant model (II + ID versus DD) and recessive model (II versus ID + DD). The Z-test was used to assess the pooled OR with the significance set at *p* < 0.05. Between-studies heterogeneity was evaluated by the Cochran C^2^-based Q test (heterogeneity was considered statistically significant if *p* < 0.10) and I^2^ statistics.^20^ An I^2^ value of 0% represents no heterogeneity; values of 25%, 50%, 75%, or more represent low, moderate, high, and extreme heterogeneity, respectively.^21^ The random-effects model (based on the DerSimonian and Laird method)^22^ was used when heterogeneity existed among studies; otherwise the fixed-effects model (based on the Mantel-Haenszel method)^23^ was applied. Departure from Hardy-Weinberg equilibrium (HWE) in healthy subjects was examined using the Chi-square test, and a *p*-value < 0.05 was considered significant. Furthermore, to explore the source of between-study heterogeneity, subgroup analyses by number of recurrent miscarriages (RMs) (≥ 2 or ≥ 3), ethnicity, and HWE status were performed. The one-way sensitivity analyses were performed to assess the stability and liability of the meta-analysis, namely, a single study in the meta-analysis was omitted each time to reflect the influence of the individual dataset to the pooled OR. Publication bias was examined by using a funnel plot and Egger's linear regression, and a *p* < 0.05 was considered significant.^24^ ^25^ All the statistical analyses were performed using the Comprehensive Meta-Analysis (CMA) software version 2.2 (Biostat, USA). All *p*-values were two-tailed with a significant level set at 0.05.

## Results

### Characteristics of Eligible Studies

**Fig. 1:**
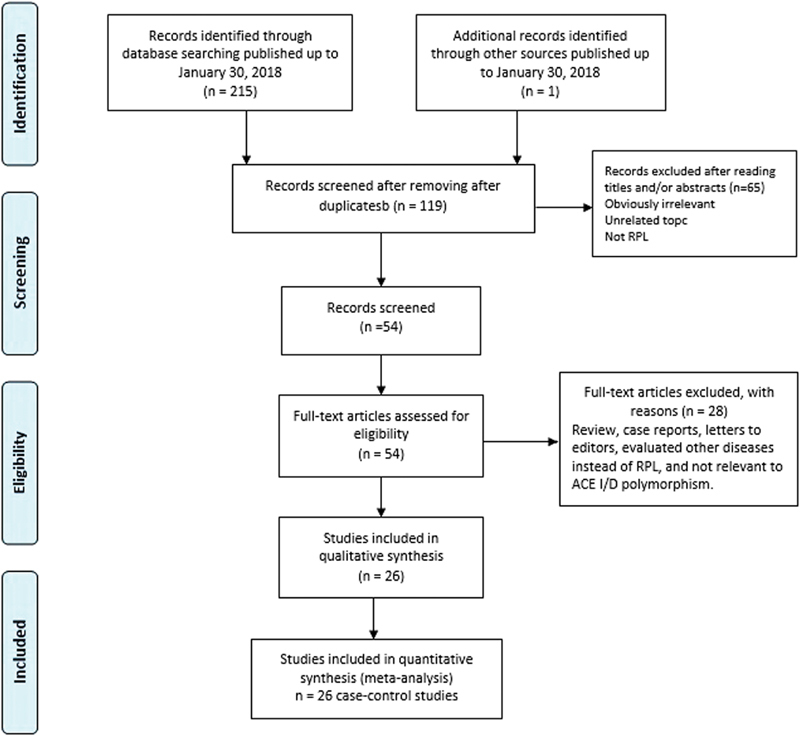
The study selection and inclusion process.

The study selection and inclusion processes are shown in Fig. 1. After a comprehensive literature search, a total of 216 publications were identified. Of these studies, x 97 were excluded in the first screening as duplicates or not relevant, leaving 119 studies for further selection. Among the remaining studies, 93 articles were excluded because they were review articles, letters to editors, previous meta-analyses, not relevant to ACE I/D polymorphism, not case-control studies, evaluated other diseases instead of RPL, and case reports. Finally, a total of 26 case-control studies with 3,140 RPL cases and 3,370 controls were included in the present meta-analysis.^14^ ^15^ ^25^ ^26^ ^27^ ^28^ ^29^ ^30^ ^31^ ^32^ ^33^ ^34^ ^35^ ^36^ ^37^ ^38^ ^39^ ^40^ ^41^ ^42^ ^43^ ^44^ ^45^ ^46^ ^47^ ^48^ The characteristics of the selected studies are summarized in Table 1. Of these, 10 case-control studies involving 1,391 cases and 1,292 controls were conducted in Caucasians,^25^ ^26^ ^28^ ^31^ ^36^ ^37^ ^40^ ^41^ ^44^ ^48^ 10 case-control studies in the west-Asians,^15^ ^27^ ^29^ ^32^ ^33^ ^39^ ^42^ ^45^ ^47^ with 984 cases and 1,108 cases, 4 case-control studies in the East-Asians,^14^ ^30^ ^34^ with 670 cases and 880 controls, 1 study involving 55 cases and 50 controls in Latinos,^43^ and 1 study with 40 cases and 40 controls in Africans.^46^ The countries of these studies included Italy, Germany, Iran, USA, India, China, Gaza, Korea, Turkey, Poland, Saudi Arabia, Sudan, Slovenia, Mexico, and Greece. All the genotype distributions of controls were in agreement with HWE for ACE I/D polymorphism except for six studies.^15^ ^28^ ^29^ ^32^ ^36^ ^45^ Therefore, 20 of 26 case-control studies were defined as high-quality studies (Tables 1, 2 and 3).

**Table 2:** Main characteristics of studies included in this meta-analysis

First Author (Year)	Country (Ethnicity)	RM (no.)	Case/Control	Cases					Controls					MAFs	HWE	Quality
				Genotype			Allele		Genotype			Allele				
				DD	ID	II	D		DD	ID	II	D	I			
Fatini et al. (2000)^26^	Italy (Caucasian)	≥3	59/70	28	21	10	77	6	20	30	20	70	70	0.500	0.231	6
Buchholz et al. (2003)^27^	Germany (Caucasian)	≥2	184/127	59	83	42	201	7	30	71	26	131	123	0.484	0.179	7
Soltan Ghoraei et al. (2007)^28^	Iran (West-Asian)	≥2	120/112	38	62	29	128	7	25	47	22	116	108	0.484	0.992	7
Goodman et al. (2008)^29^	USA (Caucasian)	≥2	120/84	34	55	31	123	6	28	34	40	74	94	0.558	0.001	6
Vettriselvi et al. (2008)^30^	India (West-Asian)	≥2	104/120	23	39	42	85	6	27	38	55	92	148	0.616	≤0.001	6
Pan (2009)^31^	China (East-Asian)	≥2	190/193	29	100	61	158	7	23	89	81	135	251	0.650	0.847	7
Bukreeva et al. (2009)^32^	Germany (Caucasian)	≥2	314/553	90	164	58	346	8	159	254	140	572	534	0.482	0.059	8
Al Sallout and Sharif (2010)^33^	Gaza (West-Asian)	≥3	100/100	49	9	42	140	6	54	12	34	142	58	0.400	≤0.001	6
Bagheri et al. (2010)^34^	Iran (West-Asian)	≥3	50/63	17	26	7	60	5	24	27	12	75	51	0.404	0.380	5
Choi et al. (2011)^35^	Korea (East-Asian)	≥3	126/251	41	50	35	132	6	44	130	77	218	284	0.565	0.391	6
Zhang et al. (2011)^14^	China (East-Asian)	≥2	127/132	21	49	57	91	6	8	34	90	50	214	0.810	0.064	6
Aarabi et al. (2011)^36^	Iran (West-Asian)	≥3	58/94	19	30	14	62	5	25	47	22	97	91	0.484	0.992	5
Corbo et al. (2011)^37^	Italy (Caucasian)	≥2	18/74	12	6	0	30	4	18	22	34	58	90	0.608	0.001	4
Ozdemir et al. (2012)^38^	Turkey (Caucasian)	≥2	327/106	121	145	61	387	8	21	51	34	93	119	0.561	0.812	8
Poursadegh Zonouzi (2013)^39^	Iran (West-Asian)	≥2	89/50	23	31	35	77	5	7	28	15	42	58	0.580	0.135	5
Kim et al. (2014)^40^	Korea (East-Asian)	≥2	227/304	34	110	83	178	8	52	148	104	252	356	0.585	0.957	8
Yalcintepe et al. (2015)^41^	Turkey (Caucasian)	≥2	20/22	9	7	4	30	5	8	9	5	25	19	0.431	0.435	5
Shakarami et al. (2015)^15^	Iran (West-Asian)	≥2	100/100	34	60	6	128	5	52	48	0	152	48	0.240	0.001	5
Kurzawińska et al. (2016)^42^	Poland (Caucasian)	≥2	152/180	40	80	32	160	6	52	84	44	188	172	0.477	0.384	6
Al-Mukaynizi et al. (2016)^43^	Saudi Arabia (West-Asian)	≥3	61/59	40	18	3	98	6	32	25	2	89	29	0.245	0.272	6
Hussian et al. (2016)^44^	Sudan (African)	≥3	40/40	23	14	3	60	6	37	3	0	77	3	0.037	0.805	6
Pereza et al. (2016)^45^	Slovenia (Caucasian)	≥3	149/149	43	75	31	161	7	55	62	32	172	126	0.422	0.071	7
Fazelnia et al. (2016)^46^	Iran (West-Asian)	≥2	100/100	29	40	31	98	6	44	33	23	121	79	0.395	0.001	6
López-Jiménez et al. (2016)^47^	Mexico (Latinos)	≥3	55/50	10	34	11	54	6	10	19	21	39	61	0.610	0.154	6
Heidari et al. (2017)^48^	Iran (West-Asian)	≥3	202/210	51	102	49	204	8	70	99	41	239	181	0.431	0.573	8
Chatzidimitriou et al. (2017)^49^	Greece (Caucasian)	≥2	48/27	21	23	4	56	6	8	10	9	26	28	0.518	0.179	6

Abbreviations: HWE, Hardy-Weinberg equilibrium; MAF, minor Allele frequency; RM, recurrent miscarriage.

**Table 3:** Results of meta-analysis for angiotensin-converting enzyme deletion/instertion polymorphism and recurrent pregnancy loss

Subgroup	Genetic model	Type of model	Heterogeneity		Odds ratio				Publication Bias	
			I^2^ (%)	P_H_	OR	95% CI	Z-_test_	P_OR_	P_Beggs_	P_Eggers_
Overall (*n* = 26)	I versus D	Random	80.49	≤0.001	0.538	0.451–0.643	−6.857	≤0.001	0.964	0.934
	II versus DD	Random	55.47	0.001	0.766	0.598–0.981	−2.109	0.035	0.980	0.991
	ID versus DD	Random	60.21	≤0.001	0.937	0.752–1.168	−0.580	0.562	0.535	0.649
	II + ID versus DD	Random	73.79	≤0.001	0.882	0.687–1.133	−0.981	0.327	0.385	0.507
	II versus ID + DD	Random	57.96	≤0.001	0.809	0.658–0.994	−2.013	0.044	0.441	0.618
RM no.										
≥2 (*n* = 16)	I versus D	Random	82.65	≤0.001	0.479	0.383–0.600	−6.427	≤0.001	0.558	0.433
	II versus DD	Random	61.52	≤0.001	0.709	0.520–0.966	−2.182	0.029	0.710	0.457
	ID versus DD	Random	46.08	0.020	0.939	0.755–1.167	−0.570	0.569	0.091	0.125
	II + ID versus DD	Random	72.93	≤0.001	0.825	0.618–1.101	−1.306	0.191	0.091	0.113
	II versus ID + DD	Random	66.47	≤0.001	0.754	0.574–0.990	−2.030	0.042	0.650	0.921
≥3 (*n* = 10)	I versus D	Random	73.55	≤0.001	0.656	0.459–1.012	−2.937	0.086	1.000	0.401
	II versus DD	Random	52.03	0.027	0.911	0.612–1.355	−0.459	0.646	1.000	0.967
	ID versus DD	Random	70.88	≤0.001	1.029	0.672–1.577	0.132	0.895	0.474	0.598
	II + ID versus DD	Random	73.33	≤0.001	1.055	0.706–1.577	0.261	0.794	1.000	0.611
	II versus ID + DD	Random	33.60	0.139	0.932	0.699–1.244	−0.478	0.633	0.720	0.861
By Ethnicity										
Caucasian (*n* = 10)	I versus D	Random	79.11	≤0.001	0.753	0.574–0.988	−2.050	0.040	0.076	0.049
	II versus DD	Random	56.28	0.019	0.640	0.440–0.930	−2.342	0.019	0.251	0.194
	ID versus DD	Random	58.75	0.013	0.866	0.630–1.192	−0.883	0.377	0.348	0.196
	II + ID versus DD	Random	73.93	≤0.001	0.643	0.439–0.943	−2.262	0.024	0.076	0.064
	II versus ID + DD	Random	49.54	0.044	0.661	0.492–0.888	−2.746	0.006	0.465	0.357
West-Asian (*n* = 10)	I versus D	Random	67.24	0.001	0.706	0.557–0.895	−2.878	0.004	0.591	0.821
	II versus DD	Fixed	14.57	0.309	1.226	0.950–1.583	1.569	0.117	0.858	0.842
	ID versus DD	Fixed	46.75	0.051	1.144	0.923–1.418	1.231	0.218	0.073	0.043
	II + ID versus DD	Fixed	41.65	0.080	1.361	1.122–1.650	3.128	0.002	0.107	0.041
	II versus ID + DD	Fixed	0.00	0.569	1.215	0.978–1.511	1.756	0.079	0.720	0.322
East-Asian (*n* = 4)	I versus D	Random	86.95	≤0.001	0.348	0.228–0.532	−4.884	0.096	0.289	0.130
	II versus DD	Random	73.96	0.009	0.574	0.307–1.072	−1.741	0.082	0.308	0.129
	ID versus DD	Random	63.20	0.043	0.709	0.426–1.180	−1.323	0.186	0.734	0.746
	II + ID versus DD	Random	72.80	0.012	0.626	0.359–1.090	−1.654	0.098	0.734	0.442
	II versus ID + DD	Random	75.49	0.007	0.713	0.460–1.106	−1.509	0.131	0.308	0.201
By HWE Status										
Not deviated (*n* = 20)	I versus D	Random	69.23	≤0.001	0.847	0.720–0.997	−2.000	0.046	0.704	0.797
	II versus DD	Random	40.95	0.036	0.693	0.545–0.881	−2.994	0.003	1.000	0.885
	ID versus DD	Random	61.17	≤0.001	0.856	0.666–1.100	−1.213	0.225	0.761	0.752
	II + ID versus DD	Random	69.41	≤0.001	0.857	0.657–1.116	−1.145	0.252	1.000	0.944
	II versus ID + DD	Random	47.51	0.013	0.787	0.643–0.964	−2.316	0.021	0.595	0.503
Deviated (*n* = 6)	I versus D	Random	85.14	≤0.001	0.901	0.559–1.451	−0.430	0.667	1.000	0.128
	II versus DD	Random	70.76	0.004	1.063	0.528–2.141	0.171	0.864	0.707	0.801
	ID versus DD	Random	32.83	0.190	1.296	0.895–1.875	1.374	0.169	0.008	0.002
	II + ID versus DD	Random	82.65	≤0.001	0.909	0.474–1.744	−0.286	0.775	0.060	0.014
	II versus ID + DD	Random	77.03	0.001	0.866	0.431–1.740	−0.404	0.686	0.452	0.931

Abbreviations: HWE, Hardy-Weinberg equilibrium; RM, recurrent miscarriage.

### Quantitative Synthesis

**Fig. 2:**
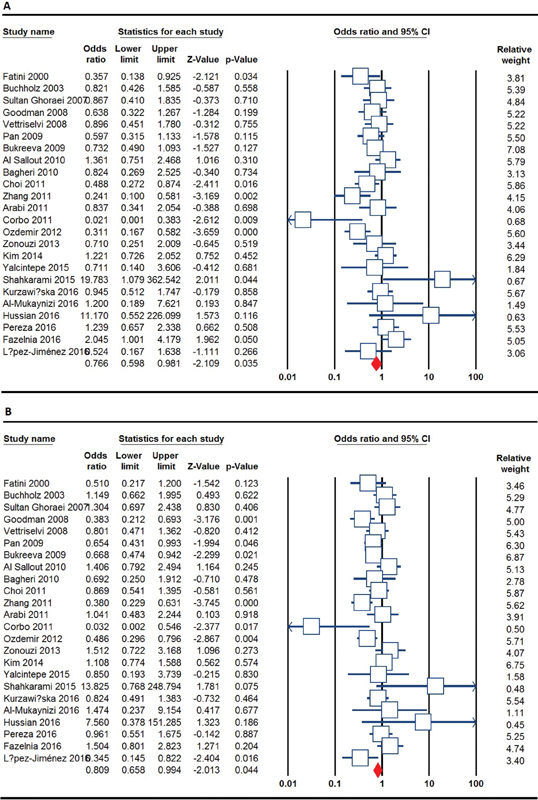
Forest plot of the association between ACE I/D polymorphism and RPL. (**A**) represents the homozygote model (II versus DD); (**B**) represents the recessive model (II versus ID + DD).

Table 2 listed the main results of the meta-analysis of ACE I/D polymorphism and RPL risk. When all the eligible studies were pooled into the meta-analysis of ACE I/D polymorphism, a significant association was found between ACE I/D polymorphism and RPL under the allele model (I versus D: OR = 0.538, 95% CI = 0.451–0.643, *p* ≤ 0.001), the homozygote model (II versus DD: OR = 0.766, 95% CI = 0.598–0.981, *p* = 0.035) (Fig. 2A) and recessive model (II versus ID + DD: OR = 0.809, 95% CI = 0.658–0.994, *p* = 0.044) (Fig. 2B).

The studies were further stratified on the basis of number of RM, ethnicity and studies HWE status. When stratified by number of RM, a significant association between ACE I/D polymorphism and increased risk of RPL was detected in the group of studies with ≥ 2 pregnancy loss under the allele model (I versus D: OR = 0.479, 95% CI = 0.383–0.600, *p* ≤0 .001), the homozygote model (II versus DD: OR = 0.709, 95% CI = 0.520–0.966, *p* = 0.029) and the recessive model (II versus ID + DD: OR = 0.754, 95% CI = 0.574–0.990, *p* = 0.042), but not in studies with ≥ 3 RM. When stratified by ethnicity, a significant association was established between ACE I/D polymorphism and increased risk of RPL among Caucasians (allele model: I versus D: OR = 0.753, 95% CI = 0.574–0.988, *p* = 0.040; homozygote model: OR = 0.640, 95% CI = 0.440–0.930, *p* = 0.019; dominant model: II + ID versus DD: OR = 0.643, 95% CI = 0.439–0.943, *p* = 0.024; and recessive model II versus ID + DD: OR = 0.661, 95% CI = 0.492–0.888, *p* = 0.006) and West-Asians (dominant model: II versus ID + DD: OR = 1.361, 95% CI = 1.122–1.650, *p* = 0.002). In contrast, no significant association was observed in the East-Asian subgroup under any of the genetic models (Table 3). Subgroup analysis of studies by HWE status showed that there was a significant association between ACE I/D polymorphism and increased risk of RPL under the homozygote model (II versus DD: OR = 0.693, 95% CI = 0.545–0.881, *p* = 0.003) and the recessive model (II versus ID + DD: OR = 0.787, 95% CI = 0.643–0.964, *p* = 0.021) in studies in accordance with HWE.

### Sensitivity Analyses

Sensitivity analysis was conducted to confirm the stability and liability of the meta-analysis by sequential omission of each eligible study. The results showed that the significance of the OR was not materially changed by any single study (data not showed), indicating the stability of our results. In addition, after the removal of studies with low quality (departure from the HWE), the corresponding pooled ORs were not significantly changed.

### Test of Heterogeneity

As shown in Table 3, there was a significant moderate to high heterogeneity among these studies for ACE I/D polymorphism in overall comparisons under all five genetic models, that is, allele (I^2^ = 80.49%, P_H _≤ 0.001), homozygote (I^2^ = 55.47%, P_H _≤ 0.001), heterozygote (I^2^ = 60.21%, P_H _≤ 0.001), dominant (I^2^ = 73.79%, P_H _≤ 0.001) and recessive model (I^2^ = 57.96%, P_H _≤ 0.001). Then, we assessed the source of heterogeneity by subgroup analyses. The I^2^ decreased obviously and the *p* value exceeded 0.05 among West-Asian studies under four genetic models, namely, homozygote (I^2^ = 14.57%, P_H _= 0.0309), heterozygote (I^2^ = 46.75%, P_H _= 0.051), dominant (I^2^ = 41.65%, P_H _= 0.080) and recessive model (I^2^ = 0.00, P_H _= 0.569), indicating that these studies were the major source of heterogeneity. However, we found that studies conducted in Caucasians and East-Asians, number of RM, and studies' quality did not contribute to heterogeneity (Table 3).

### Publication Bias

**Fig. 3:**
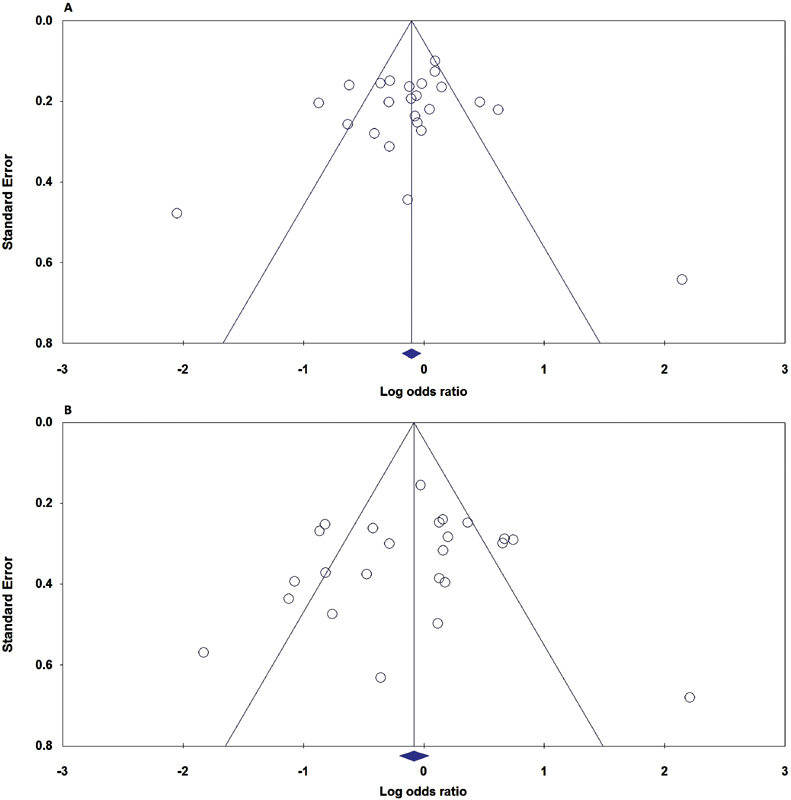
Funnel plot for publication bias in the meta-analysis of the ACE I/D polymorphism and RPL. (**A**) represents the allele model (I versus D); and (**B**) represents the dominant model (II + ID versus DD).

Publication bias of the selected articles was assessed by the funnel plot and Egger's linear regression. The shape of the funnel plot did not reveal any evidence of obvious asymmetry (Fig. 3A and B). Similarly, no evidence of publication bias was observed by the Egger's test (Table 3).

### Minor Allele Frequency (MAF)

The minor allele frequencies (MAFs) of the ACE I/D polymorphism by ethnicity are presented in Tables 1 and 2. The allele and genotype distributions of ACE I/D polymorphism exhibited ethnic variations. The ACE I allele frequency in the Caucasian, West-Asian and East-Asian populations were 51.50% (42.20–60.80%), 42.80% (24.0–61.60%), and 68.75% (56.50–81.0%), respectively. Therefore, the ACE I allele frequency in East-Asian populations was higher than in the other populations (Fig. 3).

## Discussion

The present meta-analysis was conducted to systematically identify the association between ACE I/D polymorphism with risk of RPL. In the present meta-analysis, a significant association of the ACE I/D polymorphism with risk of RPL was found under the homozygote and recessive genetic models, and in subgroup analysis, among the Caucasian and West-Asian populations. However, for East-Asians, the results indicated that the ACE I/D polymorphism was not associated with increased risk of RPL. The inconsistent results between East-Asians on subgroup analysis and pooled estimates may be caused by genetic diversity and environmental factors among different ethnicities. Furthermore, as RPL is a multifactorial condition, beside genetic factors, internal and external factors play a major role in RPL etiology. Therefore, this discrepancy might be due to other factors, such as untreated hypothyroidism, uncontrolled diabetes mellitus, certain uterine anatomic abnormalities, and antiphospholipid antibody syndrome (APS). Moreover, stratified analysis according to number of RM revealed a significantly increased risk of RPL with the ACE I/D polymorphism in those studies with ≥ 2 RM, but not in studies with ≥ 3 RM. This finding is possible because the number of studies with ≥ 2 RM included to the meta-analysis was higher than the number of studies with ≥ 3 RM. Two previous meta-analyses by Su et al^16^ and Yang et al^17^ also estimated the association between ACE I/D polymorphism and risk of RPL. Their results have confirmed that ACE I/D polymorphism is associated with the risk of RPL in overall estimates. However, their result was basically inconsistent with the present meta-analysis results, which show that ACE I/D polymorphism may not contribute to the susceptibility of RPL in Caucasians.

To further interpret the present meta-analysis results, it is necessary to clarify the difference between this meta-analysis and the previous one. More recently, Su et al^16^ performed a meta-analysis including 11 studies with 1,275 RPL cases and 2,049 controls; the study discussed the association between ACE I/D polymorphism and the risk of RPL.^16^ Their results showed a significant association between ACE I/D polymorphism and risk of RPL. In addition, their results suggested the ACE I/D polymorphism might not increase the risk of RPL in Caucasian and non-Caucasian populations. However, they only enrolled 11 case-control studies and discussed the RPL risk only under 2 dominant and recessive genetic models. Hence, it may significantly affect their overall results and subgroup results. By contrast, the present meta-analysis included 26 relevant case-control studies with higher numbers of the cases and controls and discussed the RPL risk under all 5 genetic models. In addition, we evaluated the association by quality of studies, while the prior meta-analysis did not conduct any study quality assessment. Besides, the meta-analysis by Yang et al,^17^ in 2012, reported that the ACE I/D polymorphism is associated with an increased risk of RPL in Asians and not in Caucasians, but their meta-analysis only included 9 studies with 1,264 RPL cases and 845 controls. To the best of our knowledge, the present meta-analysis was the most comprehensive meta-analysis on the association of ACE I/D polymorphism and the risk of RPL, which can provide results with greater statistical power. Moreover, with newly added case-control studies, we performed subgroup analyses to further interpret the results.

Between-study heterogeneity is a common and potential problem when interpreting the results of all meta-analyses, which should be explored in the meta-analysis.^50^ ^51^ ^52^ There are several factors responsible for such heterogeneity, such as the diverse genotype distribution of ACE I/D polymorphism in different ethnicity, sample sizes, genetic backgrounds for cases and controls, diversity in study designs, inclusion criteria, and genotyping methods.^53^ In the current study, significant heterogeneity was found in the association of ACE I/D polymorphism with RPL risk under all genetic models. Therefore, we have performed meta-regression and subgroup analyses to explore the sources of between-study heterogeneity. The results suggested that ethnicity was not the source of heterogeneity. Sensitivity analysis revealed that the removing of any single study did not have significant impact on the overall meta-analysis estimate. Moreover, the funnel plot did not reflect considerable asymmetry and the Egger's test also indicated no obvious publication bias. All these made the present meta-analysis results reliable to some extent.

The present meta-analysis had several strengths. Most importantly, it the biggest, most comprehensive and most recent meta-analysis of the association between ACE and the risk of RPL. Therefore, it was more powerful than previous cohort and case-control studies. Even though there were 26 case-control studies in our meta-analysis, its limitations should be pointed out. First, the number of studies was moderate, especially in subgroup analyses, thus limiting the interpretation of results in our analysis. Second, the study population in our meta-analysis focused on Caucasians and Asians. In the present meta-analysis, the sample size and numbers of studies in African groups and mixed groups were not adequate to evaluate any association. Thus, the conclusion may not be suitable for Africans. Second, there was significant between-study heterogeneity in all genetic models. Heterogeneity is a problem that may affect the precision of overall results. Third, the meta-analysis was based on data that had not been adjusted for the main confounding variables, such as age, gestation age at miscarriage, number of previous abortions, environmental factors and so on, which might have caused serious confounding bias. To provide a more reliable estimation of the association, more studies with a better design and a larger sample size are needed. Finally, gene–gene, gene–environment, or even different polymorphism loci of the ACE gene were not fully addressed in the meta-analysis due to lack of relevant data.

## Conclusion

In conclusion, although there was significant heterogeneity in the studies included, our meta-analysis suggests that ACE I/D polymorphism is associated with increased risk of RPL, especially in the Caucasian and West-Asian populations. However, more studies, well-designed and with a large sample, are needed to give a more reliable estimation of the association between ACE I/D polymorphism and the risk of RPL.
